# Comparing COVID-19 metaphors in Chinese and English social media with critical metaphor analysis

**DOI:** 10.3389/fpsyg.2023.1198265

**Published:** 2023-06-01

**Authors:** Qingshu Xu

**Affiliations:** ^1^School of Aeronautics, Shandong Jiaotong University, Jinan, China; ^2^Foreign Studies College, Hunan Normal University, Changsha, China

**Keywords:** CMA, MIPVU, metaphors, social media, COVID-19

## Abstract

Metaphors extracted from COVID-19-related online texts offer a unique lens for examining how individuals perceive the pandemic. Users from distinct linguistic backgrounds may select varying source domains to discuss COVID-19, with these choices influenced by multiple factors. Utilizing Critical Metaphor Analysis (CMA) theory and employing the Metaphor Identification Procedure VU (MIPVU), this study conducts a comparative analysis of Chinese and English COVID-19-related metaphors derived from social media platforms, specifically Twitter and Weibo. The findings reveal both commonalities and distinctions between the metaphors employed in Chinese and English texts. Commonalities encompass the widespread use of war and disaster metaphors in both sets of texts. Distinctions are characterized by a higher prevalence of zombie metaphors in English texts and classroom metaphors in Chinese texts. These similarities and differences can be attributed to varying socio-historical factors, as well as the active choices of users to express their values and judgments.

## Introduction

Since the inception of the COVID-19 pandemic, individuals worldwide have become increasingly familiar with various emerging concepts related to the novel coronavirus, such as the pandemic itself, pandemic prevention and control measures, pandemic development, and the lifestyles adopted under COVID-19. These concepts have gradually permeated people’s daily lives, primarily through metaphors, particularly conceptual metaphors. Numerous researchers have investigated COVID-19-related metaphors present in government-issued official documents, concentrating on the distributional characteristics of these metaphors and the rationale behind the selection of their source domains.

Social media users tend to use metaphors to express their emotions, experiences, and opinions, while governmental reports tend to use metaphors to inform policy decisions and guide public health responses. Both types of discourse play important roles in shaping public perceptions of the pandemic, and the metaphors used in each can have significant effects on how people understand and respond to the crisis. Nonetheless, it has been observed that there is a relative dearth of research focusing on COVID-19-related metaphors employed in online social platforms where internet users more actively express their understanding of the pandemic. Language usage in social media texts significantly differs from that in official documents. Farzindar and Inkpen (2018) posit that in the social media context, every user has their metaphorical “microphone” and communicates using individualized language, consequently creating a unique communication pattern. Previous studies on metaphors in social media texts have demonstrated that their source domains tend to be more culture-oriented or history-oriented (Ellis and Ryan, 2003; Guo et al., 2013; Gómez-Adorno et al., 2016; Törnberg and Törnberg, 2016; Best and Shelley, 2018).

The primary objective of this paper is to conduct a comparative analysis of COVID-19-related metaphors utilizing texts acquired from both Chinese and English social media platforms to discern the differences and commonalities between them. Firstly, textual materials are extracted from Twitter and Weibo, which are, respectively, among the most prominent social media platforms within the English and Chinese-speaking communities. Secondly, this study endeavors to identify metaphors in the collected textual materials based on the Metaphor Identification Procedure VU (MIPVU; Steen et al., 2010). In the third segment, this investigation compares the metaphors originating from both platforms in terms of differences and similarities, employing Critical Metaphor Analysis (CMA) to ascertain the underlying reasons for the metaphors’ usage. Examining COVID-19-related metaphors in social media texts enables a deeper exploration of the metaphors employed by users of both languages, as well as the cultural and historical context that informs their usage.

## Literature review

### Conceptual metaphor theory and critical metaphor analysis

Conceptual Metaphor Theory (CMT), introduced by Lakoff and Johnson (1980), posits that the core of metaphorical understanding lies in cognitive processes where one concept is comprehended through another. This entails understanding a target domain, which may be abstract, elusive, or difficult to grasp, through the source domain, which is familiar, concrete, and easily understood (Evans and Melanie, 2006). Lakoff (1993) further argue that metaphors are not just rhetorical devices, but they are essential to how we think and reason. They provide examples of how we talk about abstract concepts such as time, argument, and ideas using metaphors that draw on more concrete experiences. For instance, the metaphor “ARGUMENT IS WAR” informs the way we talk about disagreements and debates, as we use expressions such as “attack his position” or “defend my argument.” CMT has been used to explain a variety of phenomena, including emotion, morality, and politics. Some researchers apply CMT to emotion metaphors, arguing that emotions are conceptualized through metaphors such as “love is a journey” or “happiness is up” and that emotion metaphors are widely used in the context of the pandemic (Hendricks et al., 2018; de Saint et al., 2021). Similarly, researchers use CMT to explore the moral concepts that underlie political discourse, arguing that metaphors such as “the nation is a family” and “the market is a natural force” shape our understanding of political issues (Shimko, 1994; Paris, 2002; Musolff, 2016).

Critical Metaphor Analysis (CMA) is a research methodology that combines the analysis of metaphors with critical discourse analysis (Charteris-Black, 2004). CMA originates in the early 1990s, when scholars in various fields begin to question the ways in which metaphors are being used to represent and interpret social and political issues (Musolff, 2016, 2022). This leads to the development of a framework for analyzing the way that metaphors are used in political discourse, media representations, and everyday communication. According to Charteris-Black (2019), CMA is concerned with the analysis of the ways in which metaphors are used to construct meaning in discourse. The focus is on the linguistic form and social context of metaphors, as well as their underlying conceptual mappings. CMA involves a critical examination of the ideological implications of metaphors, with a particular focus on the ways in which they serve to legitimize or challenge power relations (Musolff, 2016, 2022). CMA contends that metaphor is a highly influential form of discourse. Some researchers argue that certain metaphors are deliberately chosen to impact patterns of thought and understanding among people and consequently, metaphors are frequently employed as a means to exercise power or influence (Semino et al., 2017, 2018; Charteris-Black, 2019; Semino, 2021).

The application of CMA has been demonstrated in a number of studies. For instance, Semino et al. (2018) use CMA to analyze the metaphors used in media representations of the Iraq War, and find that the use of war metaphors served to legitimize the war and delegitimize dissenting voices. Another application of CMA has been in the analysis of medical discourse, particularly in relation to illness and disease. For example, Deignan (2010) analyze the metaphors used in media representations of the SARS outbreak, and find that the use of disease metaphors served to construct a sense of threat and danger, as well as to reinforce existing cultural stereotypes about Asian people.

Mio (1997) highlights the prevalence of metaphors in political discussions, while Bougher (2012) observes that metaphors can shape public perception and judgment in the context of political events. For instance, during the U.S. presidential election, Donald Trump likened Hillary Clinton to a criminal and vehemently exclaimed, “Lock her up.” This potent metaphor swiftly gains traction and spread. Various researchers have applied CMA to examine the metaphorical construction of China’s image in the U.S. mainstream media. They conclude that these media outlets predominantly portray China as an adversary and competitor by carefully selecting source domains for their metaphors (Liu and Li, 2022).

Researchers (Foust and Murphy, 2009; Arminen and Auvinen, 2013) employ CMA to investigate ecological discourse metaphors within a self-compiled corpus and posit that the underlying expectation of these metaphors is to establish a balanced and harmonious relationship between economic development and the construction of an ecological civilization. By scrutinizing the utilization of metaphors in discourse, CMA sheds light on the ideological underpinnings and power dynamics embedded in various forms of communication.

### Previous studies on Covid-19 related metaphors

The application of metaphor studies in the analysis of COVID-19-related texts has seen a considerable expansion in recent years. Researchers have primarily concentrated on various media sources disseminating COVID-19 information, which are released by the governments of various countries. These sources include national documentaries showcasing the battle against COVID-19, white papers, and news reports. Some findings indicate that the Chinese government predominantly employed war metaphors, organism metaphors, and architectural metaphors in its campaign against the pandemic (Gui, 2021). Moreover, some scholars have scrutinized the metaphorical discourse surrounding the novel coronavirus in Western media from the perspective of CMA (Durgun et al., 2022; Alkhammash, 2023; Liu and Li, 2023).

A significant area of debate among researchers is the appropriateness of employing war metaphors extensively. Some contend that the misuse of war metaphors has led to the disregard of elements of shared concern and mutual assistance in epidemic prevention and control measures (Sabucedo et al., 2020; Isaacs and Priesz, 2021). In contrast, others justify the use of war metaphors, asserting that they neither evoke negative feelings nor effects. These proponents argue that, as integral components of social culture, war metaphors facilitate comprehension and expression of the concepts concerning the pandemic in everyday communication (Castro Seixas, 2020; Panzeri et al., 2021).

Aside from the war metaphor, researchers have also explored alternative source domains in COVID-19-related metaphors, such as ecological metaphors and the “COVID-19 IS FIRE” metaphor (Augé, 2021). Semino (2021) posits that the “COVID-19 IS FIRE” metaphor employed in COVID-19-related texts is more conducive to effective communication and discussion than the war metaphor. This burgeoning field of study underscores the importance of understanding the various ways metaphors shape public perception and discourse during unprecedented global events, such as the COVID-19 pandemic. In accordance with the findings of Belli and Alonso (2021), it has been observed that the emotional state of individuals in light of the COVID-19 pandemic can be categorized into three distinct stages. The initial stage is characterized by a sense of “indifference and curiosity,” followed by a period of “sadness” and, ultimately, a phase of “suspended mourning.” In response to these emotions and the accompanying demands for expression, netizens have resorted to utilizing metaphors on social media platforms as a coping mechanism.

### The present study

Critical Metaphor Analysis (CMA) posits that the selection of source domains in metaphors is not entirely passive. Instead, it is influenced by the values of the authors. By employing metaphors, authors aim to express and propagate specific viewpoints. The metaphors used in social media platform texts also serve as a reflection of netizens’ understanding and values. Despite its significance, few previous study has systematically analyzed the COVID-19-related metaphors utilized in social media platforms. This research seeks to bridge this gap by comparing the metaphors employed by Chinese-speaking and English-speaking netizens.

Such a comparison not only facilitates the exploration of the similarities and differences in the cognitive patterns of the two groups but also aids in understanding the culture-cognition mechanisms underlying the perception of COVID-19 by the two netizen cohorts. This is achieved by analyzing the rationale behind the selection of source domains in the metaphors. Consequently, this paper aims to address the following research questions:

1. What are the similarities and differences in COVID-19-related metaphors employed by Chinese-speaking and English-speaking netizens on social media platforms?
2. What factors contribute to the selection of source domains in the metaphors used by these two groups of netizens?
3. How do the identified metaphors provide insights into the culture-cognition mechanisms that shape the perception of COVID-19 by Chinese-speaking and English-speaking netizens?

By addressing these research questions, the study aims to contribute to the understanding of the role of metaphors in shaping public discourse and perceptions during a global crisis, such as the COVID-19 pandemic.

## Methodology

### Data

In addressing research questions 1 and 2, this paper employs Python web scraping codes to collect textual data from Twitter containing the hashtag “#COVID19” during the period of February 7, 2022 to February 13, 2022. A total of approximately 70,000 tweets are gathered, encompassing around 1,200,000 words. Upon completion of data collection, an initial data cleansing and filtering process was executed, which involved the removal of emojis, hashtags, mentions, and user IDs. Subsequently, non-English texts, such as those in Spanish and German, are eliminated. Lastly, tweets with fewer than five English words are also discarded, primarily due to the difficulty in discerning clear metaphorical expressions within such brief texts. The final dataset retains 37,834 tweets, with a total of 600,576 words. Concurrently, this paper collects Weibo textual data containing the hashtag “#epidemic” during the same timeframe, yielding roughly 13,400 Weibo posts, with a cumulative 1,510,842 Chinese characters. Following text cleaning and segmentation, the final Weibo dataset retains 9,400 posts, with an aggregate of 609,730 Chinese characters.

### Metaphor identification

In light of the comprehensive corpus amassed, the present study employs the Metaphor Identification Procedure (MIPVU), as articulated by Steen et al. (2010), as an analytical method to scrutinize the linguistic data. MIPVU, a sophisticated metaphor recognition technique meticulously devised by Steen and his associates, encompasses six methodical steps designed to facilitate the identification and classification of metaphorical expressions: (1) assiduously perusing the text to uncover metaphor-related lexemes, (2) ascertaining the foundational lexical units within the text, (3) meticulously determining the contextual meanings of these lexical units, (4) delineating the essential meanings of the lexical units, (5) judiciously differentiating between the contextual meanings of the lexical units and their intrinsic meanings, marking them as metaphorical lexemes should disparities exist, and (6) evaluating whether the contextual word classes of the lexical units deviate from the word classes of their intrinsic meanings, and if so, judiciously categorizing them into direct metaphors, indirect metaphors, and the like, contingent upon the particular circumstances. This nuanced metaphor recognition methodology has garnered widespread adoption within the realm of scientific research. Furthermore, a multitude of researchers have undertaken copious validity assessments of the MIPVU approach, with the outcomes of these diverse validity tests consistently yielding favorable results (Steen et al., 2010).

## Results

**Figure 1:**
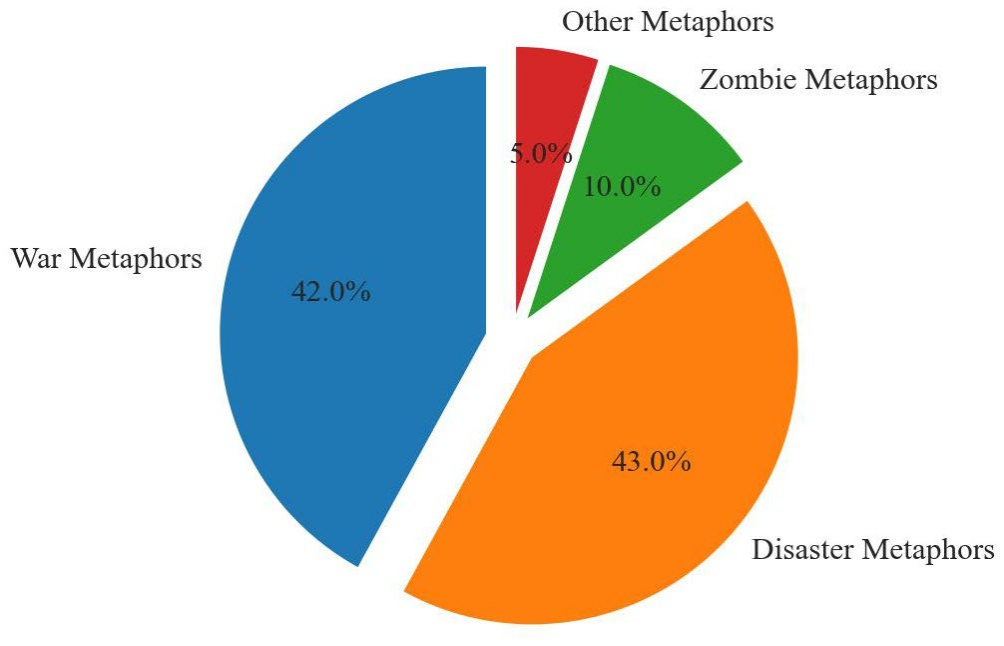
Metaphors used in English texts.

**Figure 2:**
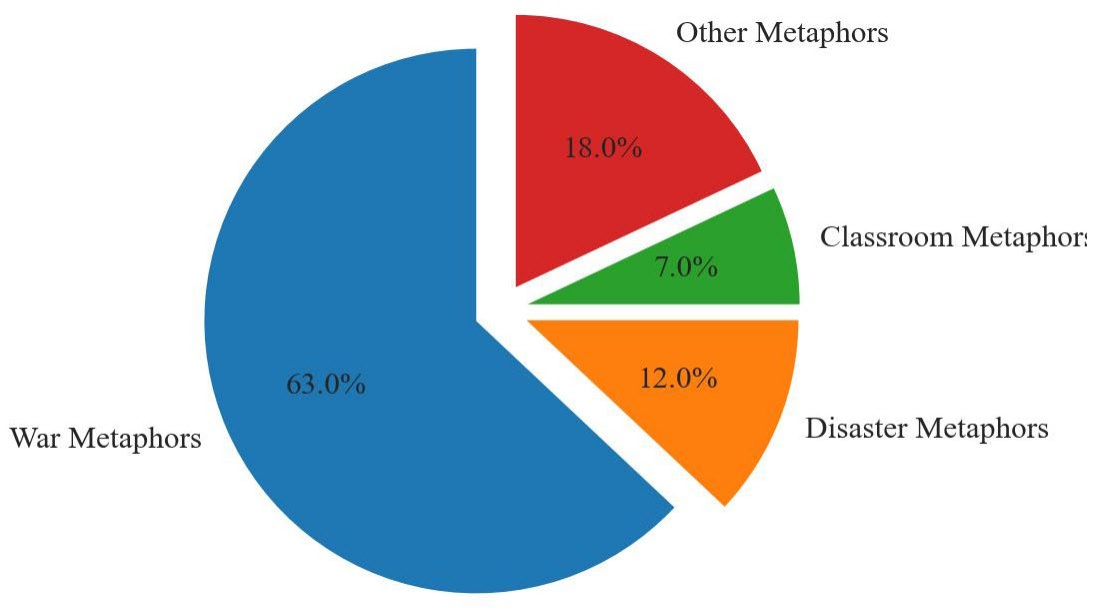
Metaphors used in Chinese texts.

Following the application of the MIPVU metaphor identification process, this study identified 13,234 English texts containing metaphorical descriptions, with a total word count of 212,105, and 3,400 Chinese texts, encompassing 220,540 Chinese characters. Concurrently, the research findings reveal that within the custom-built corpus, the metaphors commonly occurring in both Chinese and English texts pertaining to the COVID-19 pandemic primarily comprise “war metaphors” (accounting for approximately 42% of English texts and 63% of Chinese texts) and “disaster metaphors” (constituting around 43% of English texts and 12% of Chinese texts). Notably, the metaphors unique to and prominently distributed in the English texts are predominantly “zombie metaphors” (10%), whereas the metaphors exclusive to and relatively conspicuous in the Chinese texts are primarily “classroom metaphors” (7%), among others. Both the English and Chinese texts feature a smaller number of additional metaphors, which will not be discussed in this paper (5% in English texts and 18% in Chinese texts). These statistics are represented in the following two figures (Figures 1, 2).

### War metaphors

In an examination of the conceptual metaphors from an academic perspective, the “war metaphor” can be characterized as an ontological metaphor, wherein the source domain of war is predominantly employed to map adversarial target domains. Lakoff has previously posited the notion that “ARGUMENT IS WAR” (Lakoff and Johnson, 1980), which suggests that through the mechanism of conceptual metaphor, the characteristics of war are mapped from the source domain to the target domain of “argument,” thereby facilitating a more comprehensive understanding of the concept of “argument.”

The similarities between Chinese and English texts regarding the employment of war metaphors are primarily manifested in the relatively high frequency of their appearance and the close proximity of the concrete imagery chosen, such as “front line,” “weapon,” “declaration of war,” and “tactics,” among others. Four underlying reasons can be attributed to this phenomenon: firstly, both war and the COVID-19 pandemic share the outcome of death, secondly, a conspicuous adversarial nature is exhibited in both war and the pandemic, thirdly, the ubiquity of war memories in the collective human genetic makeup constitutes a crucial philosophical and cultural foundation for the widespread existence of war metaphors, and fourthly, media and government discourses have played a substantial guiding role in the application of war metaphors, as governments across the globe have extensively utilized war metaphors for social mobilization in official texts, with mainstream media also adopting war metaphors in their reportage.

Nonetheless, there are discernible distinctions in the utilization of war metaphors between the two types of texts: in the source domain of Chinese texts, a plethora of historical elements can be observed, such as expressions akin to “defensive war,” “annihilation war,” “sniper war,” “white armor,” and “donning armor for battle,” among others. The first three are mostly used to describe the battles in the People’s War of Liberation and the last two are mostly used to describe heroes like Mulan who will fight for the people and the country. These historical elements serve to explicate the proactive attitude and approach adopted by the Chinese government in combating the pandemic and to depict proactive efforts in combating the pandemic.

In the self-compiled corpus, a total of 17 key terms related to Chinese war metaphors are identified, with the top 10 most frequent terms being: “fight against the pandemic,” “resistance against the pandemic,” “front line,” “white armor,” “weapons,” “donning armor for battle,” “sniper war,” “annihilation war,” “defensive war,” and “combat.” Among these, terms such as “white armor” and “donning armor for battle” are representative of elements in ancient Chinese warfare history, while “sniper war” and “annihilation war” are emblematic of modern warfare history. These source domains are rich in connotations, evoking a multitude of associations and imaginations for the reader. Textual examples are provided below:

Text 1: 病毒内部变异出了叛徒，疫情真的有望过去了。

Translation: The virus has mutated and produced a traitor within, suggesting that the epidemic may be nearing its end.

Text 2: 此次瘟疫大战中，中国人的灵活机动完胜”联合国军”的人海战术。

Translation: In this great battle against the plague, the Chinese people's flexible and mobile strategy has decisively outperformed the human wave tactics of the “United Nations Army.”

Text 3 :一个个核酸检测采样点被迅速搭建，战役的不眠守护者筑起堡垒。

Translation: Nucleic acid testing sites were rapidly established, with the sleepless guardians of the battle constructing fortresses.

In Text 1, the Omicron variant is metaphorically portrayed as the “traitor” of the novel coronavirus. The term “traitor” frequently appears in Chinese warfare historical records, and the employment of this metaphor greatly facilitates the general public’s comprehension of the term. In Text 2, the “United Nations Army” is mentioned, a term derived from the Korean War, during which China achieved significant victories. This reference serves as an embodiment of the superiority of China’s anti-pandemic model. Text 3 posits that nucleic acid testing sites function as fortresses in the battle against the epidemic. The term “fortress” was extensively employed in the Sino-Japanese War and the People’s War of Liberation, holding significant importance in these conflicts. By likening nucleic acid testing sites to “fortresses,” the critical role they play is underscored.

Conversely, the employment of war metaphors in English texts is primarily grounded in factual descriptions, utilizing war metaphors to depict the current state of containment efforts in the context of the pandemic. Predominant examples include expressions such as “Your home is your fortress,” “medics are soldiers,” and “vaccines are stored in the arsenal.” English texts predominantly employ war metaphors for factual descriptions. In the self-compiled corpus, a total of 46 key terms related to English war metaphors were identified, with the top 10 most frequent terms being: “fight,” “shield,” “war,” “combat,” “arsenal,” “Blitz,” “front line,” “military,” “traitor.” These terms are primarily descriptive nouns and are less imbued with emotional significance. Textual examples are provided below:

Text 1: Your home is your fortress, so stay safe at home

Text 2: The un-jabbed are the ones getting sick and dying. Sort of a poetic justice. The traitors in this war against Covid19 are dying off..lol

Text 3: COVID19 best war tactic is to present itself as mild, while increasing it’s toll.

Text 4: In this war staged against humanity (Covid19), let the health guidelines take the frontline as we follow in safeguarding our health. Make masks, soap and sanitizer your weapons as well as social distancing a war tactic.

In Texts 1–4, we also observe the recurring usage of key elements within the framework of warfare, such as “allies,” “fortresses,” “traitors,” “war tactics,” and “weapons.” For example, Text 1 likens “home” to a “fortress,” encouraging residents to guard their fortress like soldiers and not to leave without proper authorization. In Text 2, individuals who have not received the vaccine are metaphorically depicted as “traitors” who have betrayed humanity’s side and joined the side of the virus. Texts 3 and 4 liken social distancing to a “war tactic” and masks, soap, and sanitizer to “weapons.”

### Disaster metaphors

In terms of disaster metaphors, both Chinese and English texts share the use of the concept of “fire.” However, the difference lies in the fact that English texts have more cases of using “forest fire” to metaphorically describe the COVID-19 pandemic. This metaphor captures the infectiousness of the COVID-19 pandemic, where even a small spark can trigger a large-scale fire. Moreover, using “forest fires” as a metaphor for the COVID-19 pandemic also has a certain basis of recognition, just as people feel the heat and high temperature in a forest fire, COVID-19 patients also experience the torture of high body temperature (Semino, 2021). At the same time, other types of disaster metaphors appear in English texts, such as “tsunami metaphors” and “storm metaphors,” which aim to awaken people’s awareness of the severity of the COVID-19 epidemic.

English texts have more disaster metaphors than Chinese texts, and there are two main reasons for this. First, compared to English-speaking countries such as the United States and the United Kingdom, the frequency of disasters such as typhoons, tsunamis, and forest fires in China is not high, and using these metaphors may not fully evoke people’s awareness of the seriousness of the COVID-19 pandemic. Second, in the “tsunami metaphor” and “storm metaphor,” the patients or viruses are compared to the “tsunami” or “storm,” while the human body or medical and health institutions are compared to the suffering side. During the time period of our sample in the self-built corpus, China did not experience large-scale epidemic outbreaks or a large influx of patients into hospitals.

Textual examples are provided below:

Text 1: If no mask or mitigation, then FL is headed towards a burn through. That’s like a forest fire that fizzles out when it runs out of wood/bushes/grass to burn.

Text 2: Charging a price for covid19 tests is like charging firefighters for water in bushfire season.

Text 3: Cases with unknown source could be “a smoldering forest fire”.

In Text 1, the Florida COVID-19 situation is likened to a forest fire that will burn through if people do not wear masks or take mitigation measures. The fire will fizzle out only when it runs out of wood, bushes, and grass to burn, which represents the three uninfected human populations. In Text 2, the COVID-19 tests are metaphorically compared to water for firefighters in the bushfire season, emphasizing the need for free testing. In Text 3, the cases of unknown sources of infection are compared to “smoldering forest fires.”

In Chinese texts, the disaster metaphor primarily revolves around the concept of fire. While the metaphor of “pandemic fire” is present, it occurs relatively infrequently. In Chinese texts, the COVID-19 pandemic is mainly compared to a fire, such as “rekindling the fire of the epidemic,” “tempering the iron army in the fire of the epidemic,” and “the fire of the epidemic has burned to our doorstep, but we are still not leaving.” However, there are not many metaphorical references to “forest fire” in Chinese texts, nor are there any extensions of this metaphor to concepts such as firefighters or firefighting equipment. Furthermore, disaster metaphors like “storm metaphors” or “tsunami metaphors” appear also less frequently in Chinese texts, with the closest being “a wave of the pandemic” which likens the pandemic to flood and similar uses. For example, in the text “They talk about wave after wave after wave. The words that are used to me are that it’s a continuous tsunami.,” the COVID-19 epidemic is compared to waves of tsunami-like waves of patients; and in “I think we are in the eye of the COVID19 Hurricane,” the current situation of the pandemic is likened to the eye of a typhoon, a momentary calm before the storm; and in “Another one calls the current phase: storm of infections,” patients are also compared to a storm that is hitting hospitals and other medical facilities.

Therefore, while the use of the disaster metaphor of “fire” is a commonality between Chinese and English texts, the difference lies in the frequency and variety of metaphorical references. English texts use a more extensive range of disaster metaphors to describe the pandemic, while Chinese texts primarily stick to the “fire metaphor.” The reason for this difference could be attributed to the lower frequency of natural disasters such as forest fires, tsunamis, and hurricanes in China, resulting in a lack of familiarity and resonance with these disaster metaphors in Chinese culture.

### Source domains chosen by users

In addition to war and disaster metaphors, English and Chinese texts use a large number of zombie apocalypse and classroom metaphors, respectively, to describe the COVID-19 pandemic. The zombie apocalypse metaphor reflects the helplessness and frustration of netizens in the face of the pandemic, while the classroom metaphor represents the pride and sense of achievement of the Chinese netizen community in the effectiveness of anti-epidemic policies.

### Zombie apocalypse metaphor

In the English text, the zombie apocalypse metaphor is used extensively. The term “zombie” comes from the French word “zombi,” referring to an undead body that has risen from the dead. With the increasing use of “zombie” elements in electronic games, television, and movies, a new concept of “zombie apocalypse” has emerged, referring to the gradual decline and collapse of human civilization with the influx of zombies, in which only a few surviving individuals remain. In some versions of the “zombie apocalypse,” it is caused by infection from viruses or parasites, and zombies sweep through key institutions of contemporary society such as law enforcement agencies, military organizations, and health organizations. Basic social services come to a standstill, and survivors can only scavenge for food, weapons, and basic supplies, and can only live in so-called safe zones. In the English-speaking world, with the popularity of cultural and entertainment products featuring this theme, “zombies” and the “zombie apocalypse” abound.

At the same time, there are obvious differences between “zombies” and the Chinese cultural concept of “僵尸(Jiangshi).” “Jiangshi” generally refers to resurrected corpses that are already dead, with stiff bodies, especially in Hong Kong films. However, zombies are different. In zombie films, living people may also be infected and become zombies. This is similar to the living environment of people during a viral outbreak. Faced with the COVID-19 pandemic, many people compared the world they faced to a zombie apocalypse, and this metaphor appeared more frequently in English texts than in Chinese texts.

Text 1: Anti-maskers keep spreading COVID19 like a zombie apocalypse.

Text 2: If COVID19 has taught us anything it’s that at least 1/3 the population is selfish AF and would hide infected bites in a real zombie apocalypse.

Text 3: I think what this Covid19 pandemic has proven is that humans would never win against a zombie apocalypse, people’s egos and pure stupidity would kill us all.

Within the aforementioned three texts, each has employed the concept of a “zombie apocalypse,” yet each text utilizes this concept as a metaphor for different things. In Text 1, those who oppose wearing masks are compared to zombies, while in Text 2, individuals who have been infected with the COVID-19 virus but refuse to seek treatment are compared to zombies. Finally, Text 3 compares the entirety of the COVID-19 pandemic to a zombie apocalypse. This metaphor is employed to convey a sense of hopelessness and despair felt by individuals who perceive the situation as bleak and dire. It serves as a powerful rhetorical tool to convey the gravity of the situation and the severity of the threat posed by the virus. Moreover, it allows individuals to express their fears and anxieties in a more accessible and relatable manner. However, it is important to note that the zombie apocalypse metaphor is not without its limitations. Its use may lead to the oversimplification of a complex issue and can potentially result in the propagation of misinformation. Additionally, it may trivialize the experiences of those who have been impacted by the pandemic, reducing their suffering to a mere pop culture reference. Thus, while the “zombie apocalypse” metaphor can be a powerful tool to convey meaning, its usage should be considered carefully and thoughtfully to avoid misrepresenting or trivializing the experiences of those impacted by the pandemic.

### Metaphor of “copying homework”

In Chinese texts, the metaphor of “copying homework” is frequently used to describe epidemic prevention and control. “Copying homework” refers to imitating successful pandemic prevention policies and practices of another government. However, this metaphor appears very rarely in English texts. In the following two Chinese texts, the metaphor of “copying homework” is used, in which one text criticizes South Korea’s failure in epidemic prevention, while the other text raises questions about Hong Kong’s epidemic prevention measures. This “copying homework” metaphor contains a semantic frame of classroom teaching. First of all, homework is generally a task assigned by the teacher to students after class, and “copying homework” is a process in which a student who cannot complete the task independently takes shortcuts and copies the results of another student with or without permission. The student who copies homework is generally a poor student, while the student whose homework is copied is generally a good student, and their homework is not usually publicly available for other students to copy. In our self-built corpus, we found that the subject of “copying homework” is generally a foreign government, while the object being copied is generally the government or city of China. This reflects the recognition of netizens of China’s achievements in epidemic prevention and control, as well as their concerns about the situation in other countries.

Text 1: 韩国能不能学点好，疫情防控抄作业都能抄个零分。

Translation: Can’t South Korea learn something useful for once? Even in pandemic prevention and control, they would fail miserably at copying the good practices of other countries.

Text 2: 大家说，香港这个疫情可以跟当时武汉抄作业。问题是，该怎么抄啊?土地面积差不多是武汉的1/8，日新增已经远超过武汉当时疫情的峰值，还有那么多不配合的人，而且变异毒株明显传染性远高于之前的病毒。

Translation: Everyone is saying that Hong Kong can learn from Wuhan's experience in epidemic prevention and control. The problem is, how can we learn from it? The land area of Hong Kong is only about 1/8 of Wuhan’s, and the daily increase in cases has far exceeded the peak of the epidemic in Wuhan at that time. In addition, there are so many uncooperative people, and the mutated strains of the virus have obviously higher transmissibility than the previous ones.

This “copying homework” metaphor contains a semantic frame of classroom teaching. First of all, homework is generally a task assigned by the teacher to students after class, and “copying homework” is a process in which a student who cannot complete the task independently takes shortcuts and copies the results of another student with or without permission. The student who copies homework is generally a poor student, while the student whose homework is copied is generally a good student, and their homework is not usually publicly available for other students to copy. In our self-built corpus, we found that the subject of “copying homework” is generally a foreign government, while the object being copied is generally the government or city of China. This reflects the recognition of netizens of China’s achievements in epidemic prevention and control, as well as their concerns about the situation in other countries.

## Discussion

The selection of source domains in metaphorical expressions is influenced by various factors such as social, historical, and cultural contexts. In our self-constructed corpus, both English and Chinese texts employ a plethora of war and disaster metaphors, indicating that both groups of netizens share a similar understanding of the COVID-19 pandemic. However, the specific selection of source domains differs significantly between the two groups. English-speaking netizens tend to use concepts from popular entertainment as source domains for metaphors, such as zombie metaphors, while Chinese-speaking netizens prefer to draw from historical elements, such as the “warriors in white” metaphor, which is used quite often to refer to young and versatile knights in Chinese folklores. Guan Yu, an important character from *Romance of the Three Kingdoms*, is an important example of “warrior in white.” Coincidentally, the medics combatting COVID-19 are also mostly dressed in white and they are also called metaphorically “warriors in white.” Even within the same source domain of war metaphors, the two groups exhibit significant differences in their selection. English-speaking netizens employ “blitz” metaphors extensively, a strategy used by Germany during World War II to quickly occupy multiple Western countries. In contrast, Chinese-speaking netizens frequently use metaphors such as “sniper war,” “defensive war,” and “annihilation war,” which were essential tactics used by the Chinese Communist Party during the Anti-Japanese War and the Liberation War.

The selection of a source domain in a metaphorical expression conveys a purpose and expresses a demand. In English texts, there are many reflective discourses on war metaphors, and even several academic papers that discuss the legitimacy and legality of using war metaphors (Semino et al., 2017, 2018; Panzeri et al., 2021). This indicates that people are not simply accepting the metaphors assigned by social, historical, and cultural factors, but rather actively selecting metaphors that reflect their own experiences. To some extent, the metaphors we rely on for survival have become the metaphors we choose to live by. Some metaphors survive this selection and become content at different levels of metaphors (Kovecses, 2010; Kövecses, 2013, 2017, 2020), while others are actively rejected. For example, the zombie metaphor is prevalent in English texts, but it has not been widely adopted in Chinese texts. In contrast, the classroom metaphor is widely used in Chinese texts, but has not gained widespread acceptance in English texts.

The different metaphors in English and Chinese texts illustrate the shared cognitive mechanisms and differences in cognitive mechanisms behind metaphors. The selection of source domains in metaphors is both passive and intentional. Passive selection is based on the collective memory of a group, while active selection is based on current values and judgments. Exploring the cognitive strategies, thinking patterns, and selection mechanisms behind metaphors not only helps us better understand metaphors, but also provides insights into the relationship between language, thought, and human values.

## Data availability statement

The raw data supporting the conclusions of this article will be made available by the authors, without undue reservation.

## Author contributions

The author confirms being the sole contributor of this work and has approved it for publication.

## Conflict of interest

The author declares that the research was conducted in the absence of any commercial or financial relationships that could be construed as a potential conflict of interest.

## Publisher’s note

All claims expressed in this article are solely those of the authors and do not necessarily represent those of their affiliated organizations, or those of the publisher, the editors and the reviewers. Any product that may be evaluated in this article, or claim that may be made by its manufacturer, is not guaranteed or endorsed by the publisher.
